# Identifying Pregnancies in Population‐Based Data Sources: Development and Application of the ConcePTION Pregnancy Algorithm

**DOI:** 10.1002/pds.70438

**Published:** 2026-08-09

**Authors:** Giorgio Limoncella, Anna Girardi, Claudia Bartolini, Giulia Hyeraci, Giuseppe Roberto, Olga Paoletti, Davide Messina, Felipe Villalobos, Carlo Alberto Bisacco, Jesse van den Berg, Eline Houben, Katia Santacà, Ylenia Ingrasciotta, Gianluca Trifirò, Giorgia Pellegrini, Valentina Ientile, Vjola Hoxhaj, Carlos Durán, Judit Riera‐Arnau, Patricia García‐Poza, Mar Martín‐Pérez, Ana Llorente‐García, Lucia Cea‐Soriano, Consuelo Huerta‐Álvarez, Francisco Sánchez‐Sáez, Gabriel Sanfélix‐Gimeno, Clara L. Rodríguez‐Bernal, Jérémy Jové, Marie‐Agnès Bernard, Nicolas H. Thurin, Sue Jordan, Daniel Thayer, Hywel Turner Evans, Alex‐Ioan Coldea, Marco Manfrini, Marleen van Gelder, Michele Tari, Romin Pajouheshnia, Ana Sofia Afonso Maciel, Maryline Le Noan‐Lainé, Ditte Mølgaard‐Nielsen, Marianne Cunnington, Caitlin Dodd, Leonardo Grilli, Constanza L. Andaur Navarro, Miriam Sturkenboom, Hedvig Nordeng, Rosa Gini

**Affiliations:** ^1^ Department of Statistics, Informatics and Applications “Giuseppe Parenti” University of Florence Florence Italy; ^2^ Department of Clinical and Experimental Medicine University of Pisa Pisa Italy; ^3^ ARS Toscana Florence Italy; ^4^ Fundació Institut Universitari Per a la Recerca a L'atenció Primària de Salut Jordi Gol i Gurina (IDIAPJGol) Barcelona Spain; ^5^ PHARMO Institute for Drug Outcomes Research Utrecht the Netherlands; ^6^ Department of Diagnostics and Public Health University of Verona Verona Italy; ^7^ Italian National Institute for Nuclear Physics Catania Italy; ^8^ Department of Biomedical and Dental Sciences and Morpho‐Functional Imaging University of Messina Messina Italy; ^9^ Department of Data Science & Biostatistics Julius Center, University Medical Center Utrecht (UMCU) Utrecht the Netherlands; ^10^ Division of Pharmacoepidemiology and Pharmacovigilance Spanish Agency of Medicines and Medical Devices (AEMPS) Madrid Spain; ^11^ Department of Public Health and Maternal Child Health Faculty of Medicine, Complutense University of Madrid; ^12^ Health Services Research and Pharmacoepidemiology Unit, Foundation for the Promotion of Health and Biomedical Research of Valencia Region Valencia Spain; ^13^ Network for Research on Chronicity, Primary Care, and Health Promotion (RICAPPS) Valencia Spain; ^14^ Departament of Applied Statistics and Operational Research, and Quality Universitat Politècnica de València Valencia Spain; ^15^ Bordeaux PharmacoEpi, INSERM CIC‐P 1401, Université de Bordeaux Bordeaux France; ^16^ Faculty of Medicine, Health and Life Science, Swansea University Wales UK; ^17^ Department of Medical Sciences, Centre for Clinical and Epidemiological Research Ferrara University Italy; ^18^ Pharmacoepidemiology and Drug Safety Research Group, Department of Pharmacy University of Oslo (UiO) Oslo Norway; ^19^ Caserta Local Health Unit Caserta Italy; ^20^ RTI Health Solutions Department of Epidemiology Barcelona Spain; ^21^ Global Patient Safety Eli Lilly BV Utrecht the Netherlands; ^22^ Global Patient Safety Eli Lilly Cork Ireland; ^23^ Global Patient Safety Novo Nordisk A/S Soeborg Denmark; ^24^ Analysis Group, Inc. London UK; ^25^ Panalgo Amsterdam the Netherlands

**Keywords:** ConcePTION pregnancy algorithm, diversity in data sources, early pregnancy detection, multi‐database study, pregnancy, random forest

## Abstract

**Purpose:**

In 2019, the Innovative Medicines Initiative funded the ConcePTION project to enhance monitoring of medication safety in pregnancy and breastfeeding. This paper describes how the ConcePTION Pregnancy Algorithm (PA) identified pregnancies in 10 diverse European electronic healthcare data sources and estimated their duration.

**Methods:**

Data sources from six European countries were mapped to the ConcePTION Common Data Model. Any pregnancy‐related record was retrieved from various available data banks, including birth register, primary care records, and hospital records, and reconciled into episodes of pregnancy (starting between 01/2015 and 12/2019), each with start date, end date, and type of end. A random forest model was used to estimate missing gestational ages for incomplete records. Parameters were tailored to data sources to address local variations in data availability, collection, and governance. Model performance was evaluated using cross‐validated Root Mean Squared Error (RMSE).

**Results:**

The PA identified ~2.7 million pregnancies, in over 2.2 million individuals. Most ended in live births (50%–83%), 1%–15% in elective terminations, and 4%–10% in spontaneous abortions, depending on data sources. Pregnancies with unknown type of end were also retrieved (2%–34%). Gestational age was predicted for 6%–89% of records (RMSE: 17–50 days). The median gestational age at first identified pregnancy record ranged from 47 to 280 days.

**Conclusions:**

We developed an open‐source algorithm to identify and date pregnancies, including early‐stage pregnancies with unknown end and/or ongoing at the time of data extraction. This algorithm may facilitate multinational studies, improving generation of timely real‐world evidence about use and safety of medicinal products in pregnancy.

## Plain Language Summary

Pregnant individuals are often excluded from clinical trials, which limits the availability of evidence on the safety of medicines used during pregnancy. To help address this gap, the Innovative Medicines Initiative funded the ConcePTION project, aimed at improving the monitoring and communication of medication safety during pregnancy and breastfeeding. As part of this initiative, we developed the ConcePTION Pregnancy Algorithm (PA), a tool designed to identify and estimate the duration of pregnancies using routinely collected healthcare data. The algorithm combines information from various sources, including hospital records, primary care data, and birth registers. When applied to 10 healthcare databases across six European countries, the PA identified approximately 2.7 million pregnancies between 2015 and 2019. It successfully captured early‐stage pregnancies, as well as those with unknown end or still ongoing at the time of data collection. Those cases are often overlooked by previously published algorithms for the identification of pregnancies. The PA employs machine learning techniques to estimate missing pregnancy start dates. As an open‐source tool, it can be tailored to accommodate diverse healthcare systems and governance restrictions. By enabling more complete and reliable identification of pregnancy episodes, the PA supports high‐quality research and the timely generation of evidence on medicine use and safety during pregnancy.

## Summary

- The end of a pregnancy in the early stages, such as spontaneous abortions, is more likely to go unrecorded in electronic healthcare databases compared to an end of pregnancy with longer course.
- Existing pregnancy‐finding algorithms generally rely on data recorded at the end of pregnancy, potentially introducing selection bias.
- We developed an algorithm to identify early‐stage, ongoing, and pregnancies with unknown ends, leveraging a Random Forest model to estimate pregnancy start dates when they are not directly recorded.
- The algorithm operates in a standardized yet adaptable manner across diverse data sources, while maintaining methodological transparency.
- We described and interpreted the algorithm's results across 10 diverse European data sources, varying in data availability.

## 1 Introduction

Real‐world evidence generated from routinely collected healthcare records is crucial for studying the effects of medicines used by pregnant and breastfeeding individuals. These populations are often excluded from non‐obstetric clinical trials due to logistical, ethical, and legal considerations [1], creating a significant information gap [2, 3].

In 2019, the Innovative Medicines Initiative (IMI) funded the ConcePTION project to enhance monitoring and communication of medication safety in pregnancy and breastfeeding (imi‐conception.eu). The project established a network of Data Expert and Access Partners (DEAPs) represented by European organizations with both access to large electronic healthcare data sources and expertise to re‐use data for pharmacoepidemiological purposes. One of the challenges addressed by the ConcePTION project was the development of an algorithm that could identify and date any pregnancy occurring in the underlying source population by leveraging information from any type of pregnancy‐related record in an electronic healthcare data source.

The occurrence of a pregnancy may prompt (i.e., trigger the generation of) records in one or more databanks of the same data source, for instance, birth registers, primary care records, hospital administrative records, and specialists' records, each providing complementary information [4]. Birth registers, although designed to collect detailed information on pregnancy outcomes, do not comprehensively contain all pregnancy episodes occurring in the underlying source population, as they typically only register completed pregnancies beyond a prespecified gestational age (commonly 12–24 gestational weeks) [5, 6, 7, 8, 9, 10]. Therefore, algorithms relying solely on these records miss pregnancies ending before that threshold of gestational age is attained. This is a form of left truncation, or ‘live birth bias’, a selection bias important in studies on adverse pregnancy or long‐term childhood outcomes [11]. Some previously published algorithms identify pregnancies based on records prompted by the end of gestation, such as hospital deliveries [12, 13], failing to detect ongoing pregnancies and pregnancies whose end does not receive medical attention, which is common in early miscarriages [14, 15]. These limitations reduce their utility in investigating novel therapies or vaccines, as they presuppose that the outcomes of exposed pregnancies prompt records in the data source, and such data become available for research. For example, an EMA‐founded 2021 study on COVID‐19 medication use in pregnancy [16], faced challenges: many data sources had accrued data until December 2020, but cases of COVID‐19 had mostly appeared after March 2020. Thus, first‐trimester exposures were not detected, either because pregnancies had not yet ended by the end of 2020, or because they had resulted in early pregnancy loss. Conversely, pregnancies with late miscarriage, termination or preterm birth were overrepresented, due to right truncation [17]. These limitations emphasize that pregnancy algorithms should be able to identify (a) when pregnancies end, (b) whether their end prompts records in the data source, and (c) whether the data prompted by the end of pregnancies become available for research.

This paper reports on how the ConcePTION Pregnancy Algorithm (PA) is configured to identify pregnancies from 10 diverse European electronic healthcare data sources and estimate their duration. We also discuss how the PA can be used to support pregnancy studies using other data sources.

## 2 Methods

### 2.1 Setting

The PA was developed as part of the IMI funded ConcePTION project and was utilized in several studies requested by the European Medicines Agency, namely CONSIGN (EUPAS39226) and the Lot4 retinoids‐valproate Risk Minimization Study (EUPAS31001 and EUPAS31095).

### 2.2 Algorithm Design

The PA is detailed in the publicly available Deliverable [18]. An overview is provided in Table 1, outlining the eight key steps of the PA, together with the list of types of pregnancy end used (Table 2), the application of a color‐coding system to classify pregnancy based on the availability or imputation of start and end dates (green: no imputation, yellow: start imputed, blue: end imputed, red: start and end imputed), and the use of a predictive model (Random Forest) to improve the imputation of pregnancy start dates. Briefly, all records implying an ongoing or completed pregnancy were retrieved from the data source. For each record, the pregnancy start date, end date, and type of pregnancy end were assigned using predefined rules developed a priori in collaboration with the DEAPs, based on the clinical context and expected timing of the event represented by the record. The full set of rules is reported in Table S1. Records referring to the same pregnancy were then reconciled and aggregated into pregnancy episodes. Finally, when sufficient information was available, a Random Forest (RF) model was used to estimate gestational age in days at the date the record was generated. In episodes where no record contained information on pregnancy start, the predicted gestational age was used to refine the pregnancy start date. The RF model is described in detail in the Supporting Information: Document 1. Although the TRIPOD guidelines [23] do not fully align with our study, we have aimed to remain as compliant as possible and have completed a modified TRIPOD‐AI checklist accordingly (see Supporting Information: Document 2). The output of the PA is a dataset with one record (row) per pregnancy. The algorithm was coded using R software (version ≥ 4.0.0), and data source configurations are stored in files in the R programming language. The script, the code of the descriptive analysis, and further documentation are publicly accessible in the GitHub repository [24]. The data were collected using version 5.2 of the script; at the time of writing, the most recent version available is 6 [25].

**TABLE 1:** Overview of the sequential steps of the ConcePTION pregnancy algorithm, including identification of pregnancy‐related records, assignment of start and end dates, reconciliation of records into episodes, and refinement of episode timing.

Step	Description	Details
1. Retrieval	The data source instance is searched and all records implying that the individual was pregnant on the record date are retrieved. Retrieval is based on 40 lists of diagnosis codes, on 9 lists of procedures codes, and on data source‐specific data elements that identify information as collected during or at the end of pregnancy (e.g., data elements that identify a recording of gestational age).	Deliverable: Section 2.2
2. Record labeling	For each record, we define three key elements: the type of pregnancy end, as listed in Table 2; the date of pregnancy end; the date of pregnancy start, defined as the date of the last menstrual period (LMP). For example: for records retrieved with a code in the code list *BirthNarrowLB*, (a) type of end is assigned to LB (live birth), (b) pregnancy end date is considered observed on the record date, and (c) start of pregnancy date is imputed as 280 days before the pregnancy end date.	Deliverable: Section 2.2
3. Record color coding	The identified pregnancy‐related records are color‐coded according to the availability of the start and end dates in the record itself: **Green**, if both pregnancy start and end date are recorded; **Yellow**, if pregnancy end date is recorded while pregnancy start date was imputed in step 2; **Blue**, if pregnancy start date is recorded while pregnancy end date was imputed in step 2; **Red**, if both pregnancy start date and pregnancy end date were imputed in step 2.	Deliverable: Section 2.2
4. Selection	Individuals are excluded if any of the following applies: The individual is not present in the population registrer; The individual is not within the fertile age range (12–55 years) at record date; The individual has only records of the following two categories: ○ Records of which the date is outside the observation periods of the person; ○ Records of which the context (stored in the record as per the ConcePTION CDM rules), based on DEAPs' expertise, is not specific to an ongoing pregnancy. For example, a DEAP may consider that a diagnosis code of spontaneous abortion recorded in a primary care administrative record could pertain to a follow‐up visit that occurred *after* the conclusion of a pregnancy. The algorithm would then be tailored so that a person who has only one such record is not classified as pregnant at the record date.	
5. Reconciliation	Multiple records referring to the same pregnancy are aggregated into a single pregnancy episode. The reconciliation process applies a comprehensive set of rules to systematically resolve discrepancies in start date, end date, and type of pregnancy end assigned in step 2 to records in the same pregnancy episode. The main ingredient in the reconciliation is the hierarchy of the code lists and other elements associated with the record. For example, information retrieved from a birth registry is attributed a higher value than conflicting information retrieved from a record carrying a hospital discharge diagnosis. Additionally, two specific parameters are used in this step to merge or separate pregnancies. The reconciliation process is detailed in the Deliverable. Both the hierarchy and the parameters can be tailored to the data sources.	Deliverable: Section 2.3.6
6. Prediction	We employ a random forest (RF) model to improve imputation of pregnancy start dates for each record. RF was selected because it does not rely on strong parametric assumptions and can flexibly model non‐linear relationships and interactions, making it suitable for heterogeneous multi‐database settings. The RF model was trained on pregnancy episodes with green or blue records. As predictors, we include: record type (i.e., the most detailed classification available for the record; when the record corresponds to a diagnosis, this is defined by the diagnosis code itself), record origin, maternal age at the record date, year of record registration, and the temporal distance from the oldest record within the pregnancy episode. The performance of the model is evaluated using a cross‐validated root mean squared error (RMSE), separately for yellow and red records.	Supporting Information: 1 and 2
7. Adjustment	In pregnancy episodes without a green or blue record, the pregnancy start date is defined as the weighted average of the predicted start dates of all records contributing to the pregnancy. The weights are calculated as the inverse of the variance of the record gestational age at the time of record registration, calculated on pregnancies with a green or blue record. We then apply systematic rules to adjust pregnancies with a duration inconsistent with the type of pregnancy end, or those that overlap with other pregnancies.	Supporting Information: 1
8. Wrap up	Finally, each pregnancy is stored as a record in a dataset, along with its main variables (start date, end date, type of end), as well as additional variables that track the choices made by the algorithm to create that pregnancy. The color code assigned to each pregnancy is the color of its highest record in the hierarchy of step 5. The full data model of the final output is presented in Table 2.3 in the Deliverable. The types of pregnancy end allowed by the PA are presented in Table 2. If mother–child link is available in the data source, the algorithm generates a dataset with pregnancy‐child link.	Deliverable: Section 2.3.8

*Note:* Details can be found in the relevant Deliverable [18] and in the Supporting Information.

**TABLE 2:** Types of pregnancy end assigned by the algorithm to each record, possible corresponding color codes, and examples.

Type of end (acronym)	Definition	Color code	Example
Live birth (LB)	Pregnancy ended in a live birth	Green	Record of a live birth retrieved from a birth registry
		Yellow	Record retrieved with one of the codes in the code list *BirthNarrowLB*
Stillbirth (SB)	Fetal death before or during the delivery, after 22 weeks of gestational age (24 weeks in UK) [5, 19]	Green	Record of a still birth retrieved from a birth registry
		Yellow	Record retrieved with one of the codes in the code list *Stillbirth—narrow*
Spontaneous abortion (SA)	Pregnancy loss before 22 weeks of gestational age (24 weeks in UK). Synonym: miscarriage, early fetal death [20]	Green	Record retrieved from a spontaneous abortion registry
		Yellow	Record retrieved with one of the codes in the code list *Spontaneousabortion—narrow*
Elective termination (T)	Legal termination of pregnancy/medical abortion, at any time during pregnancy [21]	Green	Record retrieved from an elective termination registry
		Yellow	Record retrieved with one of the codes in the code list *Interruption—narrow*
Ectopic or molar pregnancy (ECT‐MOL)	The fertilized egg implants outside the uterus or there is evidence of an abnormal product of conception	Yellow	Record retrieved with one of the codes in the code list *Ectopicpregnancy*
Unknown (UNK)	The imputed date of end of pregnancy is before the cutoff date of the data, therefore the pregnancy has surely ended, but the type of end could not be established	Blue	A record of gestational age in a primary care medical record recorded in January 2018 in a data source instance whose date of data extraction is 31st December 2018
		Red	Record of a procedure of amniocentesis recorded in January 2018 in a data source instance whose date of data extraction is 31st December 2018
Unfavorable Unspecified (UNF)	Pregnancy with observed end date, without live birth, but otherwise unspecified	Yellow	Record retrieved with one of the codes in the code list *Spontaneous abortion‐possible* (example: ICD10CM O05—Other abortion)
Birth unspecified (BUNSP)*	Pregnancy ended in a delivery (end of pregnancy at gestational age higher than 22 weeks) with unspecified vital status of the baby	Yellow	Record retrieved with a procedure code of Cesarean section
End Unspecified (UNSP)*	Pregnancy ended at the record date, but gestational age at the record date and status of the fetus are unspecified (possibly gestational age lower than 22 weeks)	Yellow	Record retrieved with one of the codes in the code list *End Unspecified* (example: ICD10 O73—Retained placenta and membranes without haemorrhage)
Pregnancy ongoing (ONGOING)	The estimated pregnancy end date is after the date of data extraction	Blue	A record of gestational age in a primary care medical record recorded in January 2018 in a data source instance whose date of data extraction is 31st March 2018
		Red	Record of a procedure of amniocentesis recorded in January 2018 in a data source instance whose date of data extraction is 31st March 2018
Lost to follow‐up (LOSTFU)	The imputed date of end of pregnancy is after the end of the observation period (i.e., a continuous period of inclusion in the underlying population of the data source) of the pregnant person, but before the date of data extraction	Blue	A record of gestational age in a primary care medical record recorded in January 2018 in a person who has left the underlying population of the data source on 31st March 2018
		Red	Record of a procedure of amniocentesis recorded in January 2018 in a person who has left the underlying population of the data source on 31st March 2018

*Note:* The code lists mentioned in the examples can be found in [22], alongside the corresponding detailed clinical definitions.

* These types of end are implemented in the version of the algorithm that was released after the publication of this work. In the version used in this study, ‘Birth unspecified’ and ‘End unspecified’ were treated as ‘Live birth’.

### 2.3 Data Sources

This study involves 10 data sources across six European countries: ARS, CASERTA, and FERR from Italy; BIFAP, SIDIAP, and VID from Spain; SNDS from France; SAIL from Wales (United Kingdom); PHARMO from the Netherlands; and UOSL from Norway. DEAPs contributing to this study participate either in the ConcePTION Project or in other initiatives using the ConcePTION Common Data Model (CDM) [26] for Electronic Health Records (EHR), which is used in this study. Data sources are represented as a union of data banks, which are distinct data collections covering the same underlying population. Each data source may be supported by different institutions for various purposes [26] (see also figure 1 in [27]). Data banks were grouped into 6 families (birth register, elective termination register, spontaneous abortion register, congenital anomaly register, hospital administrative records, primary care records) based on similarities in record prompts, reasons for data collection, and content. In Table 3, we describe the 10 data sources used in this study as combinations of the families of data banks. Additionally, in [28], each data bank was mapped according to the nine dimensions of the DIVERSE framework [29]: the DEAP, the underlying population, the prompts (i.e., events that generate records), content, data dictionary, time, healthcare and culture, and data quality. The DIVERSE framework, funded by the ISPE initiative, was developed to provide a structured approach for describing and assessing the heterogeneity of real‐world data sources, and it offers a standardized method of characterizing data banks in terms of their structure, content, and quality.

**TABLE 3:** Main characteristics of the data sources participating in the study, summarized based on the ConcePTION and DIVERSE frameworks, limited to the elements involved in the pregnancy algorithm.

Family of data bank	Details on prompt or content	Italy			Spain			France	UK	Netherlands	Norway
		ARS	FERR	CASERTA	BIFAP	SIDIAP	VID	SNDS	Wales SAIL	PHARMO	UOSL
Birth Register	Legal notification of EOP	After 22 weeks	After 22 weeks						After 24 weeks		After 12 weeks
	First contact with the perinatal caregiver									X	
	Clinical follow‐up					X	Of newborn				
Elective termination register		X									
Spontaneous abortion register		X									
Congenital anomaly register							X		X		
Hospital Administrative records	Discharge from hospital	X	X	X	X	X	X	X	X	X	X
	Access to the emergency room	X	X	X			X	Only > 1 day	x		
	Specialist visit (include diagnosis)			X			X		X		X
	Diagnostic tests	With no results					X	With no results	X		
Primary care	Medical records				X	X	X		X	X	
	Administrative records	community centers only									X

Abbreviation: EOP, end of pregnancy.

### 2.4 Application of the PA to the Data Sources

To implement the PA, each DEAP extracted an instance of their data source, defined as a subset used for conducting one or more studies [26], and converted it to the ConcePTION CDM version 2.2. The ConcePTION CDM ensures that the origin of each record can be retrieved during data processing. Quality control was monitored through INSIGHT Level 1 and 2 checks [30]. We included all individuals with records in the participating data sources between 1/Jan/2015 and 31/Dec/2019, with follow‐up to 31/Dec/2020, except for PHARMO, whose data availability was until 31/Dec/2019. In VID, PHARMO, and SNDS, only individuals with female gender were included. In the other data sources, gender was stored in the instance based on the value available at the time of data extraction, which may differ from the value at the time of pregnancy.

### 2.5 Data Source Tailored Variations of the Algorithm and Output Verification Process

The PA was tailored to each data source through an iterative verification process. This process involved incorporating DEAPs' expertise on local data structures, integrating their recommendations on key algorithmic choices, and implementing governance requirements while maintaining consistency across implementations. In practice, the PA was repeatedly run, and after each iteration, a sample of 40 pregnancies was extracted and manually reviewed by DEAPs. The extracted cases consisted of anonymized, structured summaries of all pregnancy‐related records contributing to each episode and did not involve review of original medical charts or any external clinical documentation. This sample was randomly drawn from pregnancies with inconsistencies in the available records (e.g., cases where two pregnancy‐related records provided conflicting end dates). Feedback from DEAPs, based on their manual assessment, was used to adjust algorithm rules and parameters described in Table 1 (table 2.4 in [18]). In four out of 10 data sources (ARS, VID, SNDS, and UOSL) this verification process led to modifications and improvements of the reconciliation rules to better reflect local recording practices. For example, we identified primary care records that systematically appeared after pregnancy and upon further investigation, we clarified that these corresponded to routine postpartum check‐ups rather than pregnancy events. As a result, we refined the algorithm to ensure these records were not used to determine the start or end date of pregnancy. This iterative process continued until DEAPs confirmed that the PA's choices accurately reflected their interpretation of their data.

### 2.6 Descriptive Analysis of Pregnancy Episodes

We described the variables in the dataset generated by the PA, including number of pregnancies (i.e., pregnancy episodes), number of records retrieved per pregnancy, gestational age at first record, distribution of types of pregnancy end, distribution of color codes, performance of the random forest model (measured by RMSE), and occurrence of male gender in pregnant individuals over the study years.

## 3 Results

**FIGURE 1:**
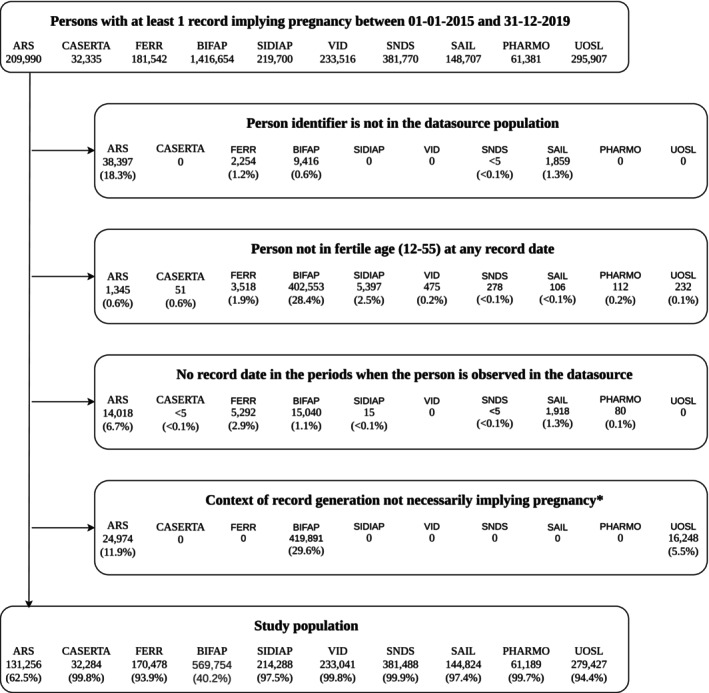
Flowchart of study population selection. Context of record generation not necessarily implying pregnancy: The records were identified in contexts not explicitly indicating pregnancy, based on DEAPs' expertise, such as diagnosis codes in primary care that may relate to post‐pregnancy follow‐ups, unrelated to an ongoing pregnancy. For BIFAP, these records mainly relate to a regular visit of a woman where her LMP is recorded by the GP, not necessarily a pregnant woman.

Among the 10 participating data sources, the algorithm identified approximately 3.2 million individuals with at least one record related to a pregnancy during the study period. The attrition diagram is presented in Figure 1. In BIFAP, more than 800 000 records out of 1.4 million were excluded—roughly half because the data entry did not necessarily imply pregnancy (e.g., last menstrual period dates recorded during routine visits do not necessarily correspond to pregnancy‐related observations), and the other half because the individuals were not of fertile age. This latter exclusion was because medical observation data related to gestational age may correspond to either the mother or the neonate at birth, as the same coding is used for both. After applying exclusion criteria, the total number of individuals included in the study exceeded 2.2 million, contributing to over 2.7 million pregnancies. Characteristics of the pregnancy cohorts are described in Table 4.

**TABLE 4:** Descriptive characteristics of the pregnancy cohorts retrieved by the algorithm between 1/1/2015 and 31/12/2019 and stratified by data source.

	ARS	CASERTA	FERR	BIFAP	SIDIAP	VID	SNDS	SAIL	PHARMO	UOSL
*Number of pregnancies*										
	164 541	37 292	208 972	729 829	264 453	329 278	485 976	166 490	73 179	393 725
*Number of records for each pregnancy*										
Mean (SD)	13.4 (10.3)	3.2 (1.6)	4.1 (2.7)	7.9 (5.5)	2.8 (1.9)	11.2 (10.9)	8.5 (4.5)	6.3 (3.2)	2.4 (2.0)	12.2 (11.3)
Median	12	3	4	7	2	9	6	6	1	10
1°–3° q	(5–19)	(2–4)	(2–6)	(3–12)	(1–4)	(2–17)	(6–11)	(5–7)	(1–3)	(5–16)
*Gestational age at first record (days)*										
Mean (SD)	59.6 (47.0)	232.1 (84.9)	194.8 (92.1)	64.0 (61.4)	186.6 (97.9)	80.4 (71.4)	100.1 (71.1)	246.7 (53.0)	138.5 (100.5)	115.2 (50.3)
Median (1°–3° q)	47 (40–57)	280 (250–280)	255 (96–273)	48 (38–60)	239 (77–278)	60 (38–87)	86 (70–97)	273 (238–280)	75 (62–271)	130 (97–136)
*Type of pregnancy end*										
LB	111 409 (67.7%)	31 000 (83.1%)	158 202 (75.7%)	434 089 (59.5%)	168 426 (63.7%)	177 247 (53.8%)	367 153 (75.5%)	161 669 (97.1%)	36 926 (50.5%)	278 355 (70.7%)
T	21 826 (13.3%)	228 (0.6%)	13 234 (6.3%)	21 296 (2.9%)	1819 (0.7%)	6120 (1.86%)	79 011 (16.3%)	NA	2267 (3.1%)	60 193 (15.3%)
SA	17 427 (10.6%)	4943 (13.3%)	23 615 (11.3%)	49 432 (6.7%)	34 029 (12.9%)	43 548 (13.2%)	23 767 (4.9%)	21 (< 0.1%)	7548 (10.3%)	40 506 (10.3%)
SB	270 (0.2%)	42 (0.1%)	273 (0.1%)	1187 (0.2%)	529 (0.2%)	882 (0.3%)	369 (0.1%)	503 (0.3%)	419 (0.6%)	855 (0.2%)
UNK	9732 (5.9%)	684 (1.8%)	9623 (4.6%)	184 663 (25.3%)	25 518 (9.6%)	89 330 (27.1%)	7590 (1.6%)	3473 (2.1%)	21 520 (29.4%)	9393 (2.4%)
ECT‐MOL	1281 (0.8%)	271 (0.7%)	2600 (1.2%)	6776 (0.9%)	3398 (1.3%)	3236 (1.0%)	6564 (1.4%)	NA	496 (0.7%)	3662 (0.9%)
UNF	147 (0.1%)	108 (0.3%)	1036 (0.5%)	26 293 (3.6%)	30 368 (11.5%)	8898 (2.7%)	1504 (0.3%)	114 (0.1%)	112 (0.2%)	757 (0.2%)
LOSTFU	2449 (1.5%)	16 (< 0.1%)	389 (0.2%)	6093 (0.8%)	366 (0.1%)	17 (< 0.1%)	18 (< 0.1%)	0	40 (0.1%)	< 5
ONGOING	0	0	0	0	0	0	0	0	3851 (5.3%)	0
*Color code*										
Green	109 567 (66.6%)	0	69 520 (33.3%)	701 (0.1%)	149 773 (56.6%)	184 867 (56.1%)	0	156 544 (94.0%)	21 040 (28.8%)	279 161 (70.9%)
Yellow	42 840 (26.0%)	36 676 (98.3%)	129 440 (61.9%)	538 372 (73.8%)	88 844 (33.6%)	55 064 (16.7%)	478 368 (98.4%)	6360 (3.8%)	26 728 (36.5%)	105 167 (26.7%)
Blue	0	0	0	79 366 (10.9%)	328 (0.1%)	1319 (0.4%)	0	0	364 (0.5%)	0
Red	12 134 (7.4%)	616 (1.7%)	10 012 (4.8%)	111 390 (15.3%)	25 508 (9.6%)	88 028 (26.7%)	7608 (1.6%)	3583 (2.2%)	25 047 (34.2%)	9397 (2.4%)
*Pregnancies recorded with male gender, per year*										
2015	100 (2.75‰)	< 5	66 (1.14‰)	130 (0.92‰)	35 (0.65‰)	*NA*	*NA*	< 5	*NA*	57 (0.68‰)
2016	173 (4.85‰)	7 (0.81‰)	76 (1.73‰)	135 (0.98‰)	33 (0.62‰)	*NA*	*NA*	< 5	*NA*	46 (0.57‰)
2017	29 (0.89‰)	< 5	70 (1.16‰)	136 (0.98‰)	36 (0.68‰)	*NA*	*NA*	< 5	*NA*	64 (0.81‰)
2018	27 (0.88‰)	< 5	67 (1.67‰)	166 (1.09‰)	36 (0.68‰)	*NA*	*NA*	< 5	*NA*	54 (0.70‰)
2019	17 (0.57‰)	< 5	67 (0.90‰)	181 (1.11‰)	38 (0.72‰)	*NA*	*NA*	< 5	*NA*	75 (0.10‰)

*Note:* Digital Health Wales prohibits reporting of elective terminations, spontaneous abortions, ectopic and molar pregnancies on the grounds that this is sensitive data. Wales is only able to report spontaneous abortions and terminations recorded in the EUROCAT register. Counts below 5 are masked. Color code indicates the availability of start and end dates: Green = start and end observed; blue = start observed, end imputed; yellow = end observed, start imputed; red = start and end imputed.

Abbreviations: ECT‐MOL, ectopic or molar pregnancy; LB, live birth; LOSTFU, lost to follow‐up; ONGOING, pregnancy ongoing; SA, spontaneous abortion; SB, stillbirth; T, elective termination; UNK, unknown.

The mean number of records contributing to each pregnancy episode ranged from 2 to 13 across the data sources. The lowest median gestational age at the first record was observed in ARS and BIFAP, at 47 and 48 days, respectively.

**FIGURE 2:**
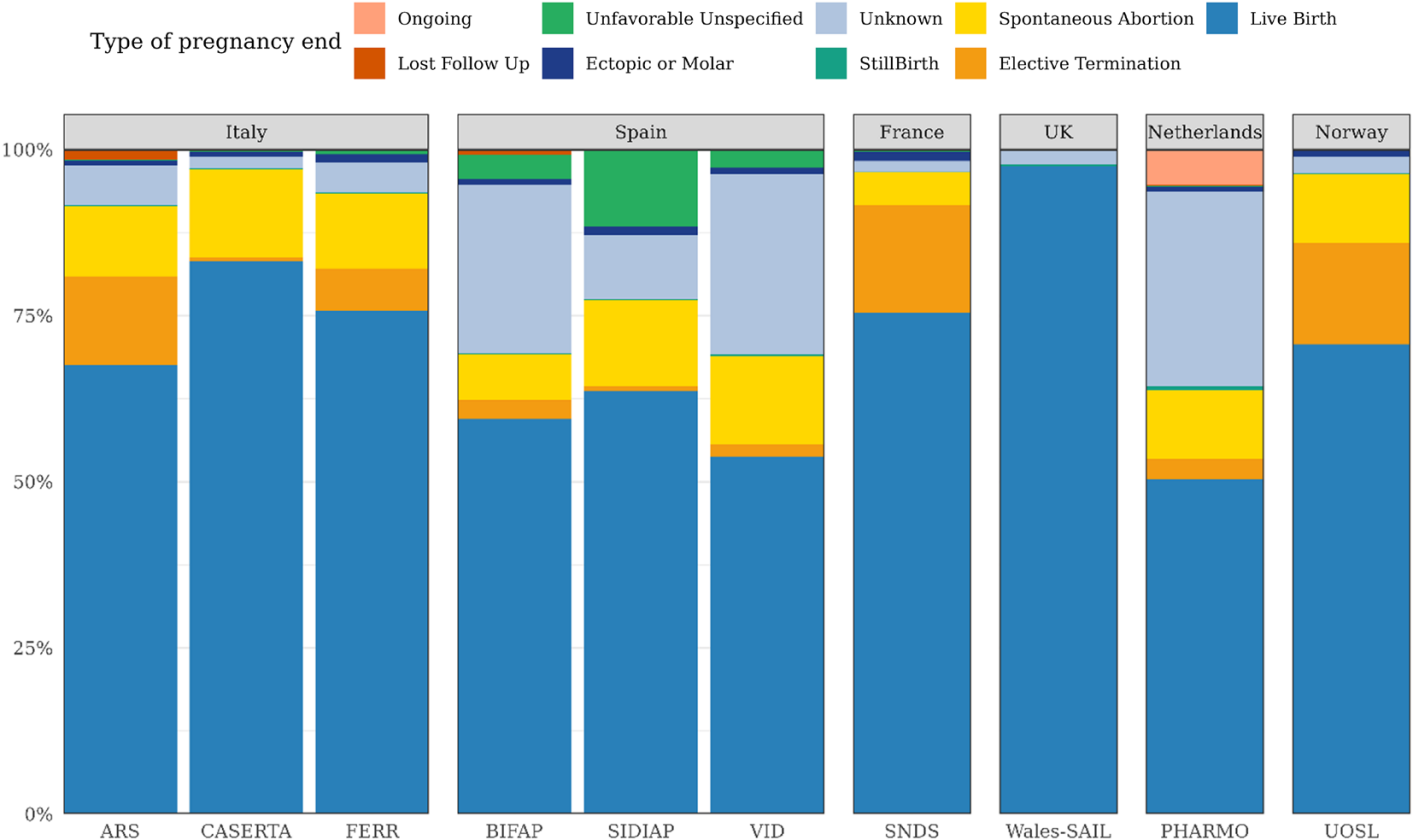
Type of pregnancy end by data source. Bars represent the proportion of pregnancy episodes by type of end (Live Birth, Stillbirth, Termination, Spontaneous Abortion, Ectopic or Molar, Unfavorable Unspecified, Unknown, Ongoing, and Lost Follow Up) within each data source.

**FIGURE 3:**
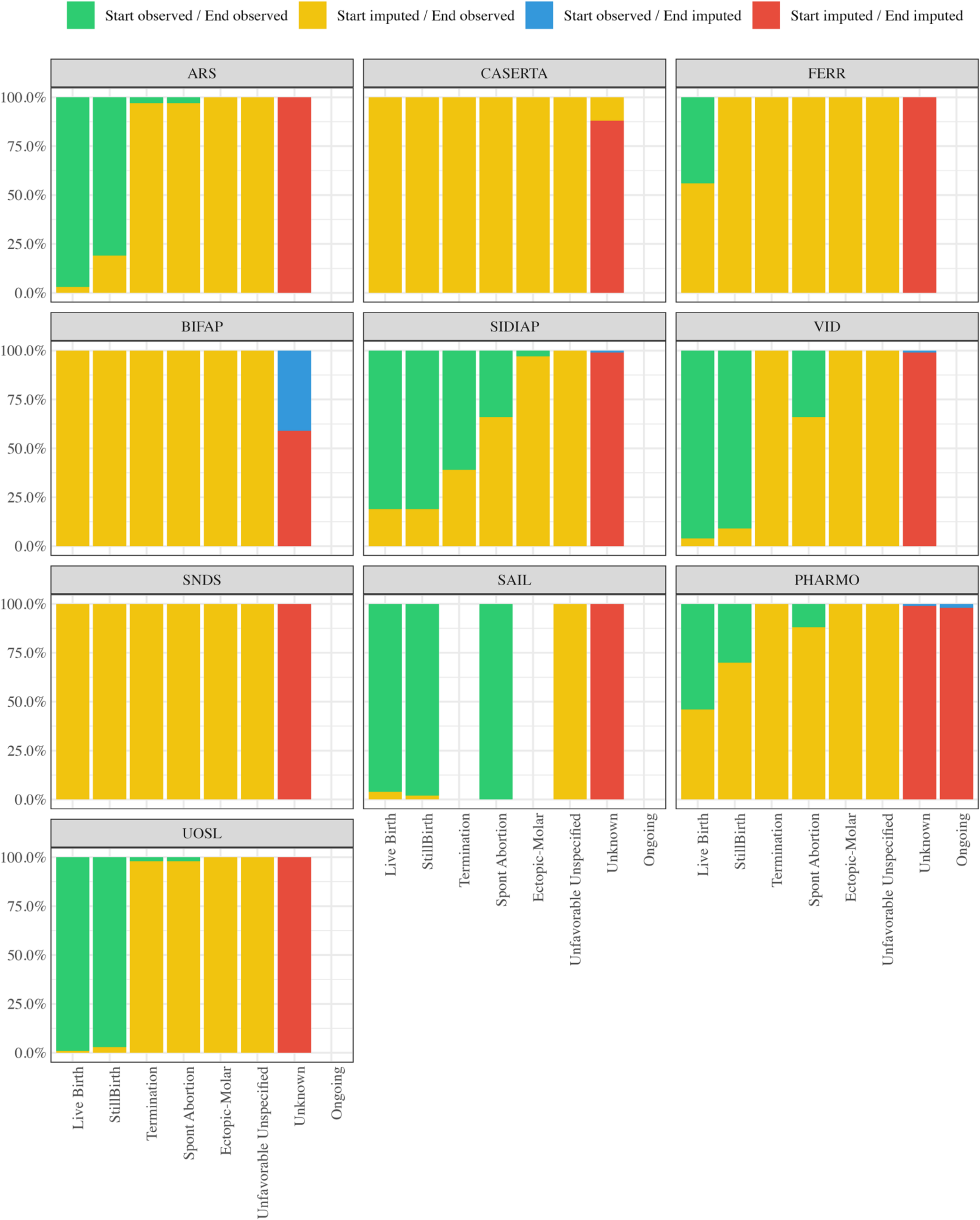
Pregnancy color code and type of pregnancy end per data source. For each data source, one bar is displayed for each type of pregnancy end. Bars are stacked to represent the proportion of pregnancy episodes by pregnancy color code (Green: Start and end observed; Blue: Start observed, end imputed; Yellow: End observed, start imputed; Red: Start and end imputed). Data sources are identified by acronym (ARS, CASERTA, FERR, BIFAP, SIDIAP, VID, SNDS, SAIL, PHARMO, UOSL). Only PHARMO identified ongoing pregnancies, as its data cut‐off was at the end of 2019. Governance rules of SAIL limited reporting of terminations and spontaneous abortion to those recorded in the EUROCAT register, therefore terminations and most spontaneous abortions were missing in SAIL. Lost follow‐up pregnancies are not presented due to small numbers.

Pregnancy cohorts varied according to the type of data availability (Table 4, Figure 2). Almost no green color‐coded pregnancies were found in data sources without birth registers (BIFAP, CASERTA, and SNDS), where yellow was the modal color code (73.8%, 98.3% and 98.4%). In data sources including a birth register, green pregnancies ranged from 28.8% (PHARMO) to 94.0% (SAIL). Blue color‐coded pregnancies almost exclusively pertained to BIFAP (10.9%), as the data source included primary care records related to visits where gestational age is recorded, but the record of a pregnancy end is missing. Red color‐coded pregnancies occurred in all data sources, from 1.7% (CASERTA) to 34.2% (PHARMO; Figure 3).

The proportion of pregnancy endings resulting in live births ranged from 50% to 83%, elective terminations from 1% to 15%, and spontaneous abortions from 4% to 10%. Pregnancies with unknown ending exceeded 25% in VID, BIFAP, and PHARMO, while SIDIAP reported 11.5% unfavorable unspecified pregnancy endings (see Table 4). Only PHARMO identified ongoing pregnancies (5.3%), as its data cut‐off was at the end of 2019 (Table 4, Figure 2). In data sources with no restrictions on gender, the proportion of pregnancies in individuals recorded as male at the data cut‐off date ranged from approximately 0.6–5 per thousand per year (Table 4).

The predictive model imputed the pregnancy start date for 6%–89% of records, depending on the data source. The root mean squared error (RMSE) ranged from approximately 17 days in ARS to 25 days in PHARMO in yellow records, and from 28 days in UOSL to 50 days in FERR in red records (Table 5).

**TABLE 5:** Cross‐validated root mean squared error (RMSE) of the Random Forest models for gestational age estimation in yellow and red episodes, by data source.

	ARS	CASERTA	FERR	BIFAP	SIDIAP	VID	SNDS	SAIL	PHARMO	UOSL
*Yellow records*										
RMSE (days)	17.0	—	17.3	24.7	22.3	23.3	—	20.1	25.1	19.2
*Red records*										
RMSE (days)	30.8	—	50.5	37.1	32.5	35.7	—	40.5	36.6	28.4

*Note:* CASERTA and SNDS do not include any pregnancy records with known start dates (i.e., blue or green records), therefore the random forest model cannot be applied.

## 4 Discussion

This paper describes how the PA was configured to identify pregnancies from 10 diverse European data sources, retrieving around 2.7 million pregnancy episodes between 2015 and 2019 from over 2.2 million individuals. Median gestational age at first record varied widely (47–280 days), reflecting the different types of data accessed, with the lowest values pertaining to ARS and BIFAP, which collect diagnostic tests during pregnancy (e.g., cardiotocography). The PA effectively handled data diversity, as shown by variation in the distribution of the color codes and types of end of pregnancies across data sources. In half of the data sources, a notable share of pregnancies had incomplete data on both start and end dates (red color‐coded: 7.4%–34.2%), or unknown type of pregnancy end (5.9%–29.4%). We attribute these patterns to several factors, that can be associated with each data source using the DIVERSE framework [29] (Table 6).

**TABLE 6:** Characteristics of data sources that have an impact on the red color‐coded pregnancies and pregnancies with unknown type of end.

Impactful characteristic	Which data source among those included in our study	Description of the impact	References
1. Health care contacts due to routinely early pregnancy visits prompt record in the data source	Data sources including records prompted in early stages of pregnancy, such as early health care contact for confirmation of pregnancy or pregnancy visits (BIFAP, SIDIAP, VID, PHARMO, ARS, SNDS, UOSL)	A share of red color pregnancies with unknown end may be attributable to early pregnancy losses.	Early pregnancy losses often go unrecorded in real‐world data [15]. Early pregnancy loss (< 12 weeks of gestation) is reported to occur in 10%–30% of pregnancies [31, 32, 33] and approximately 80% of all cases of pregnancy loss occur within the first trimester [14]
2. No birth register or birth register without mandatory notification of all births	Data sources without birth register or with a birth register based on other prompts, such as metabolic disease screening. (BIFAP, SIDIAP, VID, PHARMO)	Some pregnancies with unknown end may be attributable to live births	[28]
3. Hospitalization data banks not expected to capture all deliveries	Data banks of countries with high prevalence of home deliveries (e.g., the Netherlands), or delivery in private hospitals (e.g., Spain) (BIFAP, SIDIAP, VID, PHARMO, CASERTA, FERR)	A share of pregnancies with unknown end may be attributable to live births.	In the Netherlands, 16.3% of children born alive were born at home [34]; 18.6% of pregnancies are delivered in private hospitals in Spain [35]
4. Restrictions on elective termination reporting	Data sources where elective termination either triggers data protection regulations, raises cultural concerns, or is performed in private hospitals. The SAIL data source is subject to governance restriction for data deemed sensitive, including elective terminations, spontaneous abortions, ectopic or molar pregnancies. (BIFAP, SIDIAP, VID, PHARMO, CASERTA, FERR, SAIL)	Pregnancies ending in elective terminations may fall within red colored pregnancies with unknown end.	[36, 37]

### 4.1 Analysis According to Data Source Characteristics

Data sources including a primary care medical record data bank (BIFAP, SIDIAP, VID, and PHARMO) shared two main features: early detection of pregnancy (first quartile of gestational age between 38 and 77 days) and a significant proportion of red color‐coded pregnancies with unknown type of end, ranging from 9.6% in SIDIAP to 29.4% in PHARMO. These findings can be interpreted in light of the characteristics reported in Table 6. First, early detection increases the likelihood of identifying pregnancies that end in unnoticed early losses (first characteristic). Second, none of these data sources include a birth register or they have one without a legal mandate to record births, or they rely on hospital data, which are not expected to capture all deliveries, meaning some red‐coded pregnancies with unknown type of end may have resulted in live births (second and third characteristics). This is unlikely in VID, where a different data bank (metabolic disease registry) is designed to capture all live births. Third, elective terminations may not be fully recorded (fourth characteristic). Indeed, in these data sources we observed lower rates of elective terminations (0.7%–3.1%), compared, for example, with the French SNDS (16.3%) or the Norwegian UOSL (15.3%). For comparison, the annual rate of legally induced abortion in 2019 was higher in France (14.1 per 1000 women [38]) than in Spain and the Netherlands (7.3 and 8.6 per 1000 women, respectively [38, 39]). In SIDIAP, some elective terminations may have been categorized under ‘unfavorable’ type of end (11.5%). In pharmacoepidemiologic studies, red‐coded pregnancies with unknown ends should be carefully evaluated, considering the specific limitations and legal context of each data source.

Three other data sources (ARS, SNDS, and UOSL) also detected pregnancies early (first quartile of gestational age between 40 and 97 days) using diagnostic procedures (SNDS), primary care records (UOSL), or access to community centers (ARS). These data sources had fewer red‐coded pregnancies than primary care‐based sources, likely due to better recording of elective terminations. Moreover, in ARS and UOSL, mandatory registration into the birth registry increases the likelihood that red‐coded pregnancies with unknown type of end reflect early losses (5.9% in ARS, 2.4% in UOSL, 1.6% in SNDS).

The remaining data sources (CASERTA, FERR, and SAIL) detected pregnancies only at later stages (first quartile of gestational age from 238 days). In SAIL (Wales), governance restrictions limited data to live or stillbirths unless linked to the congenital anomaly register [36]. FERR and CASERTA relied only on hospital data alone; FERR also used a birth register subset, but no diagnostic procedures. Despite this, few red‐coded pregnancies were identified (4.6% in FERR, 1.8% in CASERTA), likely due to early losses not captured in hospital data or elective terminations. Elective termination rates were lower (6.3% in FERR, 0.6% in CASERTA) compared with ARS (13.3%), likely reflecting the absence of a termination registry and, possibly, cultural factors limiting linkage (Table 6, fourth characteristic) [37].

### 4.2 Pregnancies in Persons Recorded With Male Gender

Fewer than 2 per 1000 pregnancies involved individuals recorded as male, except in ARS. Since we did not retrieve the value of gender at the time of pregnancy, this may reflect gender transition after pregnancy. On the other hand, some cases may refer to transgender males who became pregnant after transitioning, as seen in other studies [40, 41].

### 4.3 Prediction of Start of Pregnancy

When including red and yellow pregnancies in studies on gestational age at exposure, the uncertainty in the predicted dates should be considered. In some data sources, up to 89% of pregnancy start dates were imputed, reflecting limited gestational age data. Researchers should interpret results and model performance (e.g., RMSE ranges from 17 to 51 days) with caution and consider sensitivity analyses when accuracy is crucial. Still, dates linked to diagnostic procedures (e.g., amniocentesis) are often reliable due to low variability and well‐defined timing. Patterns of missing pregnancy start and end dates are summarized in Figure S2 (Supporting Information).

### 4.4 Comparison With Other Pregnancy‐Finding Algorithms

The PA shares features with other pregnancy‐finding algorithms, such as inclusion of non‐complete pregnancies [7, 9, 10, 15, 42], those with unknown outcomes [9, 43, 44, 45], and the use of multiple data sources [46, 47, 48]. Recently, claims‐based algorithms have expanded to include records generated during gestation [6, 7, 8, 9, 10], allowing timely detection, especially for early non‐live outcomes. Campbell et al. [6] demonstrated that CPRD GOLD pregnancies with missing outcomes likely reflect real episodes, offering guidance on how to include these in studies. Studies based on U.S. and German claims data found a non‐negligible number of pregnancies with unknown ends (1.3% and 27%, respectively), highlighting the risk of underestimation of unfavorable outcomes and the generation of live birth bias [9, 43].

In a prior study using CPRD GOLD Pregnancy Register, an excessive number of pregnancy episodes arose from a heightened use of records for preconception counseling (i.e., Read code 67Iu.00 and 67It.00) without other evidence of an ongoing pregnancy [49]. The PA evolved through DEAPs' feedback: local experts verified its reliability and supported accurate interpretation of results. One example was the exclusion of diagnostic codes not necessarily implying a pregnancy at the record date.

### 4.5 Use of the PA

The PA was successfully implemented in different European studies investigating the safety of medications in pregnancy [16, 50, 51]. For instance, it enabled early identification of pregnancies affected by COVID‐19 and related treatments, which was not possible with end‐of‐pregnancy data alone [16]. Also, the PA was useful in evaluating the impact of EMA Pregnancy Prevention Programs, highlighting that excluding pregnancies with “type of end” missing can result in significant underestimation and selection bias [50].

### 4.6 Strengths and Limitations

This study applied an open‐source, R‐based pregnancy algorithm to 10 diverse data sources. The PA was highly flexible and adaptable to each source's characteristics. Although designed to be CDM‐independent (as shown in a prior project [27]), in this implementation it required conversion to the ConcePTION CDM [26]. The PA introduced several innovations. First, it processed data from different sources using a standardized framework that preserved record origin, aiding interpretation and allowing tailored selection of pregnancy characteristics [26]. Second, it employed a random forest model—shown by Zhu et al. [52] to best estimate gestational age in healthcare data—to refine pregnancy start dates. Third, it enhanced accuracy by reconciling records from the same pregnancy using specific rules (step 5, Table 1), beyond simple sorting by date and quality. Finally, it identified pregnancies ongoing at data cut‐off, as seen in Dutch PHARMO, the only source with data through end‐2019.

Limitations included potential misclassification of live births: some delivery codes (e.g., C‐sections) were labeled as live births despite unclear infant vital status, obscuring stillbirths. For instance, Welsh Wales's SAIL data indicated 0.3% stillbirths, compared with 0.4% in EUROperistat [53]. This limitation has been addressed in the latest version of the PA. Though not expected to affect live birth counts significantly, these updates are crucial for accurate stillbirth identification. Also, the inclusion of pregnancy episodes with unknown end type was intended to improve the sensitivity of the algorithm, but validation against an external reference standard was not undertaken, so some records may not represent true pregnancies. Some degree of misclassification in pregnancy identification, timing, and type of end is unavoidable as the algorithm relies on secondary healthcare data. Diagnoses and gestational age were interpreted at face value, with contradictory dates reconciled.

Representativeness of the episodes used for training the predictive model may be affected by differences in available support, individual decision‐making, and recording patterns in the data sources when an unfavorable pregnancy outcome is anticipated. This may limit the transferability of the model, potentially leading to heterogeneous prediction accuracy across data sources or across different types of pregnancy end. Reconciliation hierarchies and parameters were defined in collaboration with DEAPs based on local expertise, relevant literature, prior verifications, and clinical plausibility considerations; however, a formal quantitative sensitivity analysis comparing alternative hierarchies or thresholds was not systematically conducted across all data sources. Finally, we did not perform a formal assessment of model calibration or prediction error stratified by data‐bank family or type of pregnancy end; therefore, potential heterogeneity in predictive performance across subgroups cannot be excluded.

## 5 Conclusions

We developed an open‐source algorithm to identify and date pregnancy episodes in 10 European data sources. Unlike several existing pregnancy algorithms, it also identifies pregnancies at early stages, with unknown ends and/or still ongoing at the time of data extraction. This algorithm may facilitate multinational studies and improve generation of timely real‐world evidence about the use and safety of medicinal products in pregnancy.

## Author Contributions

ARS: Giorgio Limoncella, Anna Girardi, Claudia Bartolini, Giulia Hyeraci, and Rosa Gini contributed to the conception and design of the work, as well as to data analysis, interpretation, drafting and critical revision of the manuscript. Rosa Gini also acquired the data. Giuseppe Roberto, Olga Paoletti, Davide Messina contributed to the conception and design of the work, as well as to interpretation and critical revision of the manuscript. SIDIAP: Felipe Villalobos and Carlo Alberto Bisacco contributed to data acquisition, analysis, interpretation, drafting and critical revision. PHARMO: Jesse van den Berg contributed to data acquisition and critically revised the manuscript. Eline Houben participated in the design, data acquisition, analysis, interpretation and critical revision of the manuscript. CASERTA: Katia Santacà, Giorgia Pellegrini, and Valentina Ientile were involved in data acquisition and analysis and critically revised the manuscript. Gianluca Trifirò, Ylenia Ingrasciotta, and Michele Tari contributed to the interpretation of data and participated in the critical revision of the manuscript. BIFAP: Patricia García‐Poza and Mar Martín‐Pérez contributed to data acquisition, analysis, interpretation, and critical revision of the manuscript. Ana Llorente‐García contributed to the design and data acquisition and critically revised the manuscript. Consuelo Huerta‐Álvarez participated in the conception and design of the work and critically revised the manuscript. VID: Francisco Sánchez‐Sáez, Gabriel Sanfélix‐Gimeno, and Clara L. Rodríguez‐Bernal contributed to data acquisition and interpretation and critically revised the manuscript. Francisco Sánchez‐Sáez and Clara L. Rodríguez‐Bernal participated in data analysis. Francisco Sánchez‐Sáez contributed to drafting the manuscript. SNDS: Jérémy Jové, Marie‐Agnès Bernard, and Nicolas H. Thurin contributed to the interpretation of data and critically revised the manuscript. Jérémy Jové and Marie‐Agnès Bernard also participated in data analysis. SAIL: Sue Jordan applied for the study data and obtained all required approvals for the Wales data in this study. Thayer Daniel mapped the SAIL data onto the ConcePTION CDM. Coldea Alex‐Ioan ran and cleaned the scripts, with help from Hywel Turner Evans. Sue Jordan curated and interpreted the data, benchmarked to published data, and approved uploading of aggregated data. FERR: Marco Manfrini was involved in data acquisition, analysis, and interpretation, and critically revised the manuscript. UOSL: Hedvig Nordeng applied for the study data and obtained all required approvals for the Norwegian data in this study. Hedvig Nordeng and Vera Mitter Hayati mapped the Norwegian data onto the ConcePTION CDM. Vjola Hoxhaj and Constanza L. Andaur Navarro contributed to the interpretation of data and participated in both drafting and critical revision of the manuscript; Vjola Hoxhaj also contributed to the conception of the work. Carlos Durán, Marianne Cunnington, and Leonardo Grilli were involved in the conception of the work and critically revised the manuscript; Caitlin Dodd and Marianne Cunnington also contributed to the design. Judit Riera‐Arnau, Constanza L. Andaur Navarro, Marleen van Gelder, and Ditte Mølgaard‐Nielsen contributed to data analysis and interpretation and critically revised the manuscript. Romin Pajouheshnia and Maryline Le Noan‐Lainé contributed to the conception or design of the work, participated in data interpretation, and critically revised the manuscript. Ana Sofia Afonso Maciel contributed to data acquisition and interpretation and critically revised the manuscript. Miriam Sturkenboom contributed to the conception, design, and acquisition of data, and was involved in data interpretation, drafting, and critical revision of the manuscript.

## Funding

The ConcePTION project has received funding from the Innovative Medicines Initiative 2 Joint Undertaking under grant agreement No. 821520. This Joint Undertaking receives support from the European Union's Horizon 2020 research and innovation program and EFPIA. Some data expert and access partners participating in the CONSIGN project (EUPAS39226) and the Lot4 Retinoids‐Valproate Risk Minimization Study (EUPAS31001 and EUPAS31095), both funded by the European Medicines Agency, reused the configuration of the ConcePTION Pregnancy Algorithm developed in these projects in the present study.

## Ethics Statement

PHARMO: For the current database research with anonymous data, no ethics committee approval was required. SAIL: This study uses anonymised data held in the Secure Anonymised Information Linkage (SAIL) Databank. The SAIL Databank independent Information Governance Review Panel (IGRP) approved the study as part of project 0823, on 16th October 2020. FERR: The study was approved by the local ethical committee (approval number 593/2023/Oss/UniFe). Data were handled and stored in accordance with the General Data Protection Regulation and in agreement with the Authority for Healthcare and Welfare, Emilia Romagna Regional Health Service, Bologna, Italy. ARS: In Italy, the participation in the ConcePTION pregnancy algorithm study was included in ARS Toscana workplan and it did not require any further ethical approval. SNDS: Access to the SNDS to complete this project has been granted by the Commission nationale de l'informatique et des libertés (CNIL) in compliance with ethical and legal regulation (loi n° 2016–41 du 26 janvier 2016). UOSL: The study was approved by the Regional Committee for Research Ethics in South‐East Norway (approval number 85224) and by the Data Protection Officer at the University of Oslo (approval number 519858). Data were handled and stored in accordance with the General Data Protection Regulation VID. The project was developed in accordance with the Declaration of Helsinki, the International Guidelines for Ethical Review of Epidemiological Studies, European and Spanish regulation on biomedical research, and European regulation (General Data Protection Regulation 2016/679; GDPR‐2016) and Spanish (Organic Law 3/2018, of December 5, on the Protection of Personal Data and guarantee of digital rights; LOPDP‐2018) on protection of personal data. The project uses pseudo‐anonymized data transferred after the corresponding authorization by the Regional Health Authority (Conselleria de Sanidad Universal y Salud Pública) of the Valencia region that is responsible for the anonymization and will retain the necessary information for a possible reidentification in the cases provided by law. The project was submitted for approval by the Drug Research Ethics Committee (CEIm) of the University Hospital Clinic of Valencia. BIFAP: The use of BIFAP data for this study was covered under the approvals granted by the Scientific Committee of BIFAP (protocol references: 18/2019 and 19/2019) and the Ethics Committees (Comité de Ética de la Investigación con Medicamentos Regional de la Comunidad de Madrid and Comité de Ética de Investigación con Medicamentos del Hospital Clínico Universitario de Valencia) respectively, for two previous studies: “Impact of EU label changes and revised pregnancy prevention programme for medicinal products containing valproate: Utilization and prescribing trends” (EMA/2017/09/PE) and “Impact of EU label changes and revised pregnancy prevention programme for oral retinoid containing medicinal products: utilization and prescribing trends” (EMA/2017/09/PE), in which the use of the ConCEPTION pregnancy algorithm, the main objective of the current manuscript, was already included. Informed consent need was waved by the ethics committee given the nature of the source of data. CASERTA: The participation in the ConcePTION pregnancy algorithm study was included in the context of an agreement with Caserta LHU and it did not require any further ethical approval. SIDIAP: Code: 21/199‐PCV.

## Conflicts of Interest

Giuseppe Roberto works as a consultant for ARS Toscana, a publicly owned research center that has research contracts funded by public and private institutions, including pharmaceutical companies, and is compliant with the ENCePP Code of Conduct. Giulia Hyeraci, Olga Paoletti, Davide Messina and Rosa Gini are employed by ARS Toscana, a publicly owned research center that has research contracts funded by public and private institutions, including pharmaceutical companies, and compliant with the ENCePP Code of Conduct. Ylenia Ingrasciotta is the CEO of the academic spin‐off ‘INSPIRE srl,’ which has received funding for conducting observational studies from contract research organizations (RTI Health Solutions, Pharmo Institute N.V.) and from pharmaceutical Companies (Chiesi Italia, Kyowa Kirin s.r.l., Daiichi Sankyo Italia S.p.A.). Gianluca Trifirò participated in advisory boards and seminars as lecturer on topics not related to the paper and sponsored by the following pharmaceutical companies in the last 2 years: Eli Lilly; Sanofi; Amgen; Novo Nordisk; Sobi; Gilead; Celgene; Daiichi Sankyo, Takeda and MSD. He is also scientific coordinator of the pharmacoepidemiology team at the University of Verona and of the academic spin‐off ‘INSPIRE srl’ that carried out in the last 2 years observational studies/systematic reviews on topics not related to the content of this article and which were funded by PTC Pharmaceutics, Kyowa Kirin, Shionogi, Shire, Chiesi and Daiichi Sankyo. Judit Riera‐Arnau is a currently salaried employee at University Medical Center Utrecht, which receives institutional research funding from pharmaceutical companies and regulatory agencies, administered by University Medical Center Utrecht. All these studies follow the ENCePP code of conduct. Jérémy Jové is a researcher at Bordeaux PharmacoEpi, a research platform of the University of Bordeaux and its subsidiary the ADERA, which performs financially supported studies for public and private partners in compliance with the ENCePP Code of Conduct. Marie‐Agnès Bernard is a biostatistician at Bordeaux PharmacoEpi, a research platform of the University of Bordeaux and its subsidiary the ADERA, which performs financially supported studies for public and private partners in compliance with the ENCePP Code of Conduct. Romin Pajouheshnia is an employee of RTI Health Solutions, which is a unit of RTI International, a nonprofit organization that conducts work for government, public, and private organizations, including pharmaceutical companies. Maryline Le Noan‐Lainé is an employee and stockholder of Eli Lilly and Company. At the time of the original study, Marianne Cunnington was an employee of GSK and owned shares in GSK, which contributed person time to the ConcePTION project. Miriam Sturkenboom is head of a department that conducts research for pharmaceutical companies, using the algorithm described in this paper.

## Supporting information


**Table S1:** Rules used by the Pregnancy Algorithm (PA) to assign pregnancy start date, end date, and type of pregnancy end based on the characteristics of pregnancy‐related records. The table reports rules applied to records originating from different data banks, including registries (e.g., EUROCAT congenital anomaly registry, birth registers), hospitalization (diagnosis codes) and routine healthcare records. For each type of record, the algorithm defines the pregnancy start date and end date either directly from information available in the record or by applying predefined rules (e.g., subtracting a fixed number of days from the record date or from the end date). The columns “imputed start” and “imputed end” indicate whether the corresponding date was derived through imputation rather than directly observed in the record. The final column reports the color code assigned to the record according to the availability of start and end dates: Green (both dates observed), yellow (start date imputed), blue (end date imputed), and red (both dates imputed). Acronyms: ECT, ectopic pregnancy; LB, live birth; SA, spontaneous abortion; SB, stillbirth; T, elective termination; UNF, unfavorable unspecified pregnancy end; UNK, unknown pregnancy end.
**Figure S1:** illustration of the steps in processing pregnancy records reported in Table 1. The colors of the records represent: green (no imputation), yellow (start date imputed), blue (end date imputed), and red (both start and end dates imputed). The diamond shape indicates the record registration date, the circle represents the recorded pregnancy start date, and the star symbolizes the imputed start date predicted by the RF model. The bar length corresponds to the duration of the pregnancy.
**Figure S2:** Proportion of pregnancy episodes according to missing pregnancy start and end dates across participating databases. Acronyms correspond to the participating data sources: ARS, CASERTA, FERR, BIFAP, SIDIAP, VID, SNDS, SAIL, PHARMO, and UOSL.

**Data S1:** TRIPODAI‐checklist.

## Data Availability

All relevant data are within the paper and its Supporting Information files. Authors may not share the study data due to regulations which restrict access and distribution to those with ethical and legal permission to use the data. The study material is available to other researchers upon an application to relevant register holders. The study protocol was registered in the EUPAS Registry (EUPAS50142). All code lists and scripts can be found at https://github.com/ARS‐toscana/ConcePTIONAlgorithmPregnancies. This work was built on Section 5.4.2 of the Data Characterization Protocol of the ConcePTION Project, version 1.0. The statistical analysis plan is available at https://docs.google.com/document/d/1HzmoAOi9x9CSRZdg5qCwEHTQqMvJSthZ.
