# Supplementary material for: Identifying Pregnancies in Population‐Based Data Sources: Development and Application of the ConcePTION Pregnancy Algorithm
Source: Pharmacoepidemiol Drug Saf. 2026 Aug 9;35(8):e70438. doi: 10.1002/pds.70438 (PMC13453551; doi:10.1002/pds.70438)
Supplement: Supplementary file 2 [file PDS-35-e70438-s001.docx]

**Supplementary document 1**

**Rules used by the Pregnancy Algorithm (PA) to assign pregnancy start date, end date, and type of pregnancy end**

We reported in Table S1 the rules applied to define pregnancy end date, pregnancy start date, and type of pregnancy end of records originating from different data banks, including registries (e.g., EUROCAT congenital anomaly registry, birth registries), hospitalization (diagnosis codes) and routine healthcare records. The columns “imputed start” and “imputed end” indicate whether the corresponding date was derived through imputation rather than directly observed in the record. The final column reports the color code assigned to the record according to the availability of start and end dates: green (both dates observed), yellow (start date imputed), blue (end date imputed), and red (both dates imputed).

Table S1. Rules used by the Pregnancy Algorithm (PA) to assign pregnancy start date, end date, and type of pregnancy end based on the characteristics of pregnancy-related records. The table reports rules applied to records originating from different data banks, including registries (e.g., EUROCAT congenital anomaly registry, birth registers), hospitalization (diagnosis codes) and routine healthcare records. For each type of record, the algorithm defines the pregnancy start date and end date either directly from information available in the record or by applying predefined rules (e.g., subtracting a fixed number of days from the record date or from the end date). The columns “imputed start” and “imputed end” indicate whether the corresponding date was derived through imputation rather than directly observed in the record. The final column reports the color code assigned to the record according to the availability of start and end dates: green (both dates observed), yellow (start date imputed), blue (end date imputed), and red (both dates imputed). Acronyms: LB = live birth; SB = stillbirth; SA = spontaneous abortion; T = elective termination; ECT = ectopic pregnancy; UNK = unknown pregnancy end; UNF = unfavourable unspecified pregnancy end.

|  | **Type of pregnancy end** | **Start date** | **End date** | **Imputed start** | **Imputed end** | **Color** |
| --- | --- | --- | --- | --- | --- | --- |
| **EUROCAT - congenital anomaly registry** | | | | | | |
| record with gestational age recorded | Derived from record | Derived from record | Derived from record | no | no | Green |
| record without gestational age recorded | Derived from record | End date - 280 | Derived from record | no | yes | Yellow |
| **Other Registers (e.g., Birth registries)** | | | | | | |
| record with gestational age recorded | Derived from record | Derived from record | Derived from record | no | no | Green |
| record without gestational age recorded | Derived from record | End date - 280 | Derived from record | no | yes | Yellow |
| **Routine healtcare record (non-diagnostic code)** | | | | | | |
| Last menstrual period | UNK | From record | start date + 280 | no | yes | Blue |
| Positive pregnancy test | UNK | Record date - 30 | start date + 281 | yes | yes | Red |
| **Diagnosis codes related to end of pregnancy** | | | | | | |
| Live birth with gestational age <24 weeks | LB | Record date - 154 | record date | no | no | Green |
| Live birth at 24 weeks | LB | Record date - 168 | record date | no | no | Green |
| Live birth at 25–26 weeks | LB | Record date - 178 | record date | no | no | Green |
| Live birth at 27–28 weeks | LB | Record date - 192 | record date | no | no | Green |
| Live birth at 29–30 weeks | LB | Record date - 206 | record date | no | no | Green |
| Live birth at 31–32 weeks | LB | Record date - 220 | record date | no | no | Green |
| Live birth at 33–34 weeks | LB | Record date - 234 | record date | no | no | Green |
| Live birth at 35–36 weeks | LB | Record date - 248 | record date | no | no | Green |
| Live birth ≥37 weeks | LB | Record date - 266 | record date | no | no | Green |
| Birth (specific code list) | LB | Record date -280 | record date | yes | no | Yellow |
| Term delivery | LB | Record date -280 | record date | yes | no | Yellow |
| Preterm delivery | LB | Record date -250 | record date | yes | no | Yellow |
| Post-term delivery | LB | Record date -300 | record date | yes | no | Yellow |
| Stillbirth | SB | Record date - 280 | record date | yes | no | Yellow |
| Elective termination | T | Record date - 70 | record date | yes | no | Yellow |
| Spontaneous abortion | SA | Record date - 70 | record date | yes | no | Yellow |
| Ectopic pregnancy | ECT | Record date - 70 | record date | yes | no | Yellow |
| Possible termination | UNF | Record date - 70 | record date | yes | no | Yellow |
| Possible stillbirth | UNF | Record date - 280 | record date | yes | no | Yellow |
| Possible spontaneous abortion | UNF | Record date - 70 | record date | yes | no | Yellow |
| **Procedures codes related to end of pregnancy** | | | | | | |
| Delivery procedure (live birth) | LB | Record date -280 | record date | yes | no | Yellow |
| Delivery procedure | LB | Record date -280 | record date | yes | no | Yellow |
| Termination procedure | T | Record date - 70 | record date | yes | no | Yellow |
| Spontaneous abortion procedure | SA | Record date - 70 | record date | yes | no | Yellow |
| Ectopic pregnancy procedure | ECT | Record date - 70 | record date | yes | no | Yellow |
| **Diagnosis codes related to an ongoing pregnancy** | | | | | | |
| Gestational age <24 weeks recorded | UNK | Record date - 154 | start date + 280 | no | yes | Blue |
| Gestational age 24 weeks recorded | UNK | Record date - 168 | start date + 280 | no | yes | Blue |
| Gestational age 25–26 weeks recorded | UNK | Record date - 178 | start date + 280 | no | yes | Blue |
| Gestational age 27–28 weeks recorded | UNK | Record date - 192 | start date + 280 | no | yes | Blue |
| Gestational age 29–30 weeks recorded | UNK | Record date - 206 | start date + 280 | no | yes | Blue |
| Gestational age 31–32 weeks recorded | UNK | Record date - 220 | start date + 280 | no | yes | Blue |
| Gestational age 33–34 weeks recorded | UNK | Record date - 234 | start date + 280 | no | yes | Blue |
| Gestational age 35–36 weeks recorded | UNK | Record date - 248 | start date + 280 | no | yes | Blue |
| Gestational age ≥37 weeks recorded | UNK | Record date - 266 | start date + 280 | no | yes | Blue |
| Possible delivery record | UNK | Record date -280 | record date | yes | yes | Red |
| Record indicating ongoing pregnancy | UNK | Record date - 55 | start date + 280 | yes | yes | Red |
| Gestational diabetes | UNK | Record date - 55 | start date + 280 | yes | yes | Red |
| Fetal growth restriction | UNK | Record date - 55 | start date + 280 | yes | yes | Red |
| Pre-eclampsia | UNK | Record date - 55 | start date + 280 | yes | yes | Red |
| Pregnancy bleeding | UNK | Record date - 55 | start date + 280 | yes | yes | Red |
| **Procedures codes related to an ongoing pregnancy** | | | | | | |
| Procedure during pregnancy | UNK | Record date - 55 | start date + 280 | yes | yes | Red |
| Nuchal translucency screening | UNK | Record date - 55 | start date + 280 | yes | yes | Red |
| Amniocentesis | UNK | Record date - 55 | start date + 280 | yes | yes | Red |
| Chorionic villus sampling | UNK | Record date - 55 | start date + 280 | yes | yes | Red |
| Other pregnancy-related record | UNK | Record date - 55 | start date + 280 | yes | yes | Red |

**Developing and evaluating the performance of a multivariable prediction model of start date of pregnancy, in a population of records identifying pregnancies**

**Introduction**

We employed a predictive model to improve imputation of pregnancy start dates. The starting point is the output of step 5 in Table 1. Each record represents a pregnancy whose start date may be observed (green or blue records) or imputed (yellow or red records). Moreover, records are grouped if they are believed to represent the same pregnancy. We refer to a group of records representing the same pregnancy as a *pregnancy episode*.

To enhance the accuracy of these imputations, we implemented a random forest (RF). This method was selected due to its robust predictive performance on large datasets, computational efficiency, and capacity to handle complex, non-linear relationships among features (1). In this document we report on the development and evaluation of the predictive model.

**Methods**

The process is illustrated in Figure S1 and described below:

1. **Dataset splitting**: The dataset was divided into two subsets:

- Subgroup A: includes all pregnancy episodes with at least one blue or green record, where the start date is observed. We defined the observed start date of the episode as the start date of the green or blue record. In case of conflicting start dates recorded, the hierarchy among records was used (see step 5 of Table 1 in the paper)
- Subgroup B: includes all pregnancy episodes with no blue or green records, where the start date is imputed.

2. **Start date assignment in subgroup A and assessment of variability**: all the records of pregnancy episodes in subgroup A were assigned the observed start date of their episode. This allowed calculation of gestational age at record date for all records. Subsequently records were labeled with a *classifier*, defined as a group of records sharing the same clinical context (e.g., a classifier “postterm” including all diagnosis codes referring to post-term delivery). This allowed us to calculate the variance of such gestational age separately per classifier.
3. **Model training and evaluation**: The yellow and red records from subgroup A were used to train the RF model. The model's performance was evaluated using a cross-validated root mean squared error (RMSE).
4. **Prediction** (subgroup B): The trained RF model was applied to predict gestational age (in days) at record date, for all records in subgroup B.
5. **Pregnancy start date definition**:

- For subgroup A, the pregnancy start was confirmed as the observed date (see above).
- For subgroup B, the pregnancy start date was estimated as follows. The predicted gestational age at record date was converted into start date expressed as number of days between 1^st^ January 1970 and the predicted start date. Each record was assigned its classifier and the corresponding variance as computed in step 2. Pregnancy start date was defined as the weighted average of the predicted start dates from all records contributing to the pregnancy, with weights determined as the inverse of the variance.

6. **Postprocessing**

Based on clinical input, we assumed that gestational age at end of pregnancy must fall within the following intervals, according to the type of end:

- LB: between 147 and 310 days
- T: between 14 and 154 days
- SA: between 14 and 154 days
- UNF: between 14 and 310 days
- SB: between 154 and 310 days
- ECT: between 14 and 154 days
- LOSTFU: between 14 and 310 days
- ONGOING: between 14 and 310 days
- UNK: between 14 and 310 days

If the predicted gestational age at end of pregnancy fell outside the corresponding interval, we re-imputed it as the mean gestational age in the pregnancies of type A with the same type of end.

The covariates used in the random forest were the following:

- *Record type*: refers to the specific description of the record at the most granular aggregation level available. If the record is associated with a diagnosis code, the record type value corresponds to the diagnosis code itself.
- *Origin*: refer to the origin table name from which the record is retrieved.
- *Age*: mother's age at the record date.
- *Year*: year of record registration, used to account for potential changes in recording methods over time.
- *Distance*: distance from the date in which the oldest record belonging to the same pregnancy is recorded, in days.

By construction predictors had no missing data.

Hyperparameters were optimized by computing 5-fold cross-validated RMSE, where the folds were randomly split 80%–20%, for different setting of *Number of trees*, *Number of selected variables*, and *Variable always split* (2). The model used for prediction used the parameters combination that minimized the RMSE. We tested separately for each DEAP all combinations of the following hyperparameters value:

- *Number of trees*: 100, 500
- *Number of selected variables*: 2, 3, 4
- *Variable always split:* none, *Record type*

No independent fully held-out test sample was used in addition to the cross-validation procedure.

Figure S1: illustration of the steps in processing pregnancy records reported in Table 1. The colors of the records represent: green (no imputation), yellow (start date imputed), blue (end date imputed), and red (both start and end dates imputed). The diamond shape indicates the record registration date, the circle represents the recorded pregnancy start date, and the star symbolizes the imputed start date predicted by the RF model. The bar length corresponds to the duration of the pregnancy.

**Patterns of missing pregnancy dates across data sources**

The proportion of pregnancy episodes with missing start and/or end dates varied across databases and is reported in Figure S2. Databases such as SAIL, UOSL, and ARS showed a high proportion of episodes with no missing dates, whereas CASERTA and SNDS were predominantly composed of episodes with missing start date only. Episodes with both start and end dates missing were more frequent in PHARMO, VID, and BIFAP, while episodes with missing end date only were uncommon and mainly observed in BIFAP.

Figure S2: Proportion of pregnancy episodes according to missing pregnancy start and end dates across participating databases. Acronyms correspond to the participating data sources: ARS, CASERTA, FERR, BIFAP, SIDIAP, VID, SNDS, SAIL, PHARMO, and UOSL.

**Bibliography**

1. Wright M. N., & Ziegler, A. (2017). ranger: A Fast Implementation of Random Forests for High Dimensional Data in C++ and R. *Journal of Statistical Software*, *77*(1), 1–17. https://doi.org/10.18637/jss.v077.i01
2. Breiman L. (2001). Random forests. Machine learning, 45(1), 5-32.
